# 2-Chloro-*N*-[3-cyano-1-(3,4-dichloro­phen­yl)-1*H*-pyrazol-5-yl]acetamide

**DOI:** 10.1107/S1600536812008094

**Published:** 2012-03-24

**Authors:** Ming Li, Jing Zhu, Hong-xia Wei, Jian-qiang Wang, Cheng Guo

**Affiliations:** a College of Science, Nanjing University of Technology, Xinmofan Road No. 5 Nanjing, Nanjing 210009, People’s Republic of China

## Abstract

In the title compound, C_12_H_7_Cl_3_N_4_O, the dihedral angle between the pyrazole and benzene rings is 35.6 (3)°. In the crystal, mol­ecules are linked by N—H⋯O hydrogen bonds generating *C*(4) chains propagating in [100].

## Related literature

For background to the properties of *N*-pyrazoles, see: Liu *et al.* (2010 ▶); Zhao *et al.* (2010 ▶).
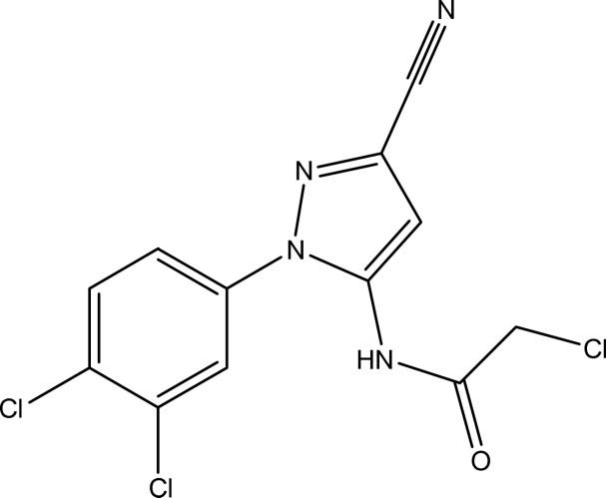


## Experimental

### 

#### Crystal data

C_12_H_7_Cl_3_N_4_O
*M* *_r_* = 329.57
Monoclinic, 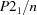

*a* = 4.6280 (9) Å
*b* = 17.245 (3) Å
*c* = 17.468 (4) Å
β = 94.04 (3)°
*V* = 1390.7 (5) Å^3^
*Z* = 4
Mo *K*α radiation
μ = 0.66 mm^−1^
*T* = 293 K
0.30 × 0.20 × 0.10 mm

#### Data collection

Enraf–Nonius CAD-4 diffractometer
Absorption correction: ψ scan (North *et al.*, 1968 ▶) *T*_min_ = 0.827, *T*_max_ = 0.937
5687 measured reflections
2564 independent reflections
1880 reflections with *I* > 2σ(*I*)
*R*_int_ = 0.040
3 standard reflections every 200 reflections intensity decay: 1%

#### Refinement

*R*[*F* ^2^ > 2σ(*F* ^2^)] = 0.042
*wR*(*F* ^2^) = 0.139
*S* = 1.01
2564 reflections
182 parameters
H-atom parameters constrained
Δρ_max_ = 0.31 e Å^−3^
Δρ_min_ = −0.25 e Å^−3^

### 

Data collection: *CAD-4 Software* (Enraf–Nonius, 1989 ▶); cell refinement: *CAD-4 Software*; data reduction: *XCAD4* (Harms & Wocadlo, 1995 ▶); program(s) used to solve structure: *SHELXS97* (Sheldrick, 2008 ▶); program(s) used to refine structure: *SHELXL97* (Sheldrick, 2008 ▶); molecular graphics: *SHELXTL* (Sheldrick, 2008 ▶); software used to prepare material for publication: *SHELXL97*.

## Supplementary Material

Crystal structure: contains datablock(s) global, I. DOI: 10.1107/S1600536812008094/hb6649sup1.cif

Structure factors: contains datablock(s) I. DOI: 10.1107/S1600536812008094/hb6649Isup2.hkl

Supplementary material file. DOI: 10.1107/S1600536812008094/hb6649Isup3.cml

Additional supplementary materials:  crystallographic information; 3D view; checkCIF report

## Figures and Tables

**Table 1:** Hydrogen-bond geometry (Å, °)

*D*—H⋯*A*	*D*—H	H⋯*A*	*D*⋯*A*	*D*—H⋯*A*
N4—H4*A*⋯O^i^	0.86	1.95	2.743 (3)	153

Symmetry code: (i) 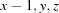.

**Fig. 1.**
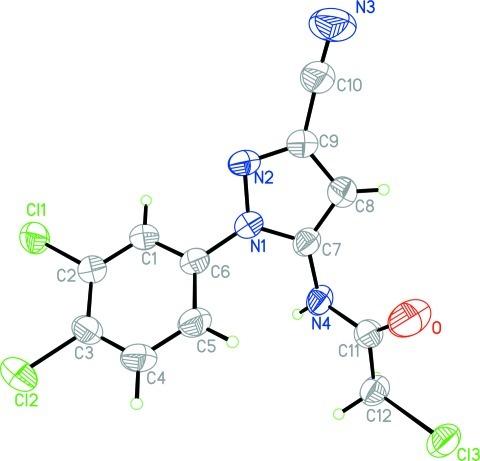
A view of the molecular structure of (I). Displacement ellipsoids are drawn at the 50% probability level.

**Fig. 2.**
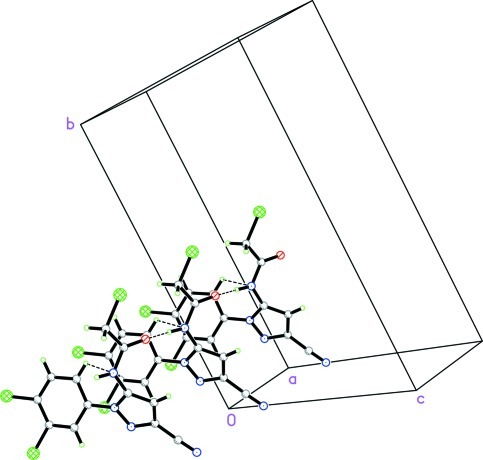
Packing diagram for (I).

## supplementary crystallographic information

### Experimental

To a stirred solution of 5-amino-1-(3,4-dichlorophenyl)-1H-pyrazole-3-carbonitrile (5 mmol) in THF (20 ml) was added 2-chloroacetyl chloride (5 mmol) dropwise at 0-5°C. After the addition, the reaction mixture was allowed to raise to room temperature and stirred for 2 h. The crude product (I) precipitated and was filterd. Pure compound (I) was obtained by crystallization from ethanol. Colourless blocks of (I) were obtained by slow evaporation of an acetone solution.

### Refinement

All H atoms bonded to the C atoms were placed geometrically at the distances of 0.93-0.97 Å and included in the refinement in riding motion approximation with U_iso_(H) = 1.2 or 1.5U_eq_ of the carrier atom.

### Crystal data

**Table:**

C_12_H_7_Cl_3_N_4_O	*D*_x_ = 1.574 Mg m^−^^3^
*M**_r_* = 329.57	Melting point: 473 K
Monoclinic, *P*2_1_/*n*	Mo *K*α radiation, λ = 0.71073 Å
*a* = 4.6280 (9) Å	Cell parameters from 25 reflections
*b* = 17.245 (3) Å	θ = 9–13°
*c* = 17.468 (4) Å	µ = 0.66 mm^−^^1^
β = 94.04 (3)°	*T* = 293 K
*V* = 1390.7 (5) Å^3^	Block, colorless
*Z* = 4	0.30 × 0.20 × 0.10 mm
*F*(000) = 664	

### Data collection

**Table:**

Enraf–Nonius CAD-4 diffractometer	1880 reflections with *I* > 2σ(*I*)
Radiation source: fine-focus sealed tube	*R*_int_ = 0.040
Graphite monochromator	θ_max_ = 25.4°, θ_min_ = 1.7°
ω/2θ scans	*h* = 0→5
Absorption correction: ψ scan (North *et al.*, 1968)	*k* = −20→20
*T*_min_ = 0.827, *T*_max_ = 0.937	*l* = −21→21
5687 measured reflections	3 standard reflections every 200 reflections
2564 independent reflections	intensity decay: 1%

### Refinement

**Table:**

Refinement on *F*^2^	Secondary atom site location: difference Fourier map
Least-squares matrix: full	Hydrogen site location: inferred from neighbouring sites
*R*[*F*^2^ > 2σ(*F*^2^)] = 0.042	H-atom parameters constrained
*wR*(*F*^2^) = 0.139	*w* = 1/[σ^2^(*F*_o_^2^) + (0.090*P*)^2^] where *P* = (*F*_o_^2^ + 2*F*_c_^2^)/3
*S* = 1.01	(Δ/σ)_max_ < 0.001
2564 reflections	Δρ_max_ = 0.31 e Å^−^^3^
182 parameters	Δρ_min_ = −0.25 e Å^−^^3^
0 restraints	Extinction correction: *SHELXL97* (Sheldrick, 2008), Fc^*^=kFc[1+0.001xFc^2^λ^3^/sin(2θ)]^-1/4^
Primary atom site location: structure-invariant direct methods	Extinction coefficient: 0.019 (3)

### Special details

**Table:**

Geometry. All esds (except the esd in the dihedral angle between two l.s. planes) are estimated using the full covariance matrix. The cell esds are taken into account individually in the estimation of esds in distances, angles and torsion angles; correlations between esds in cell parameters are only used when they are defined by crystal symmetry. An approximate (isotropic) treatment of cell esds is used for estimating esds involving l.s. planes.
Refinement. Refinement of F^2^ against ALL reflections. The weighted R-factor wR and goodness of fit S are based on F^2^, conventional R-factors R are based on F, with F set to zero for negative F^2^. The threshold expression of F^2^ > 2sigma(F^2^) is used only for calculating R-factors(gt) etc. and is not relevant to the choice of reflections for refinement. R-factors based on F^2^ are statistically about twice as large as those based on F, and R- factors based on ALL data will be even larger.

### Fractional atomic coordinates and isotropic or equivalent isotropic displacement parameters (Å^2^)

**Table:**

	*x*	*y*	*z*	*U*_iso_*/*U*_eq_	
O	0.8240 (5)	0.38138 (13)	0.35313 (18)	0.0813 (8)	
Cl1	−0.40136 (18)	0.21696 (4)	−0.00295 (5)	0.0641 (3)	
C1	−0.0120 (6)	0.23735 (15)	0.11596 (15)	0.0438 (6)	
H1A	−0.0419	0.1852	0.1266	0.053*	
Cl2	−0.3173 (2)	0.39579 (5)	−0.03563 (5)	0.0738 (3)	
N1	0.3510 (5)	0.24173 (12)	0.22197 (12)	0.0426 (5)	
N2	0.4349 (5)	0.16733 (12)	0.21185 (13)	0.0488 (6)	
C2	−0.1647 (6)	0.27227 (15)	0.05502 (15)	0.0447 (6)	
Cl3	0.69321 (17)	0.53185 (4)	0.41501 (5)	0.0602 (3)	
C3	−0.1258 (7)	0.35054 (16)	0.04024 (15)	0.0496 (7)	
N3	0.8375 (9)	0.01856 (18)	0.2981 (2)	0.0950 (11)	
N4	0.3729 (4)	0.34308 (13)	0.31669 (13)	0.0456 (6)	
H4A	0.1933	0.3562	0.3119	0.055*	
C4	0.0724 (8)	0.39217 (16)	0.08606 (17)	0.0582 (8)	
H4B	0.0990	0.4446	0.0762	0.070*	
C5	0.2322 (7)	0.35752 (15)	0.14625 (16)	0.0521 (7)	
H5A	0.3688	0.3857	0.1763	0.062*	
C6	0.1850 (6)	0.27986 (14)	0.16112 (14)	0.0412 (6)	
C7	0.4522 (5)	0.26933 (16)	0.29174 (15)	0.0425 (6)	
C8	0.6121 (7)	0.21299 (17)	0.32747 (17)	0.0541 (7)	
H8A	0.7116	0.2150	0.3756	0.065*	
C9	0.5948 (7)	0.15113 (15)	0.27610 (16)	0.0489 (7)	
C10	0.7278 (8)	0.07622 (19)	0.28682 (18)	0.0644 (9)	
C11	0.5659 (6)	0.39367 (15)	0.34757 (15)	0.0426 (6)	
C12	0.4304 (6)	0.46675 (15)	0.37652 (18)	0.0503 (7)	
H12A	0.3004	0.4534	0.4157	0.060*	
H12B	0.3171	0.4916	0.3346	0.060*	

### Atomic displacement parameters (Å^2^)

**Table:**

	*U*^11^	*U*^22^	*U*^33^	*U*^12^	*U*^13^	*U*^23^
O	0.0337 (12)	0.0558 (13)	0.154 (3)	0.0049 (10)	0.0002 (13)	−0.0304 (15)
Cl1	0.0735 (6)	0.0549 (5)	0.0603 (5)	−0.0049 (4)	−0.0212 (4)	0.0010 (3)
C1	0.0494 (16)	0.0346 (13)	0.0471 (15)	−0.0013 (12)	0.0018 (12)	0.0003 (11)
Cl2	0.0949 (7)	0.0570 (5)	0.0673 (5)	0.0099 (4)	−0.0091 (5)	0.0194 (4)
N1	0.0480 (13)	0.0351 (11)	0.0440 (12)	−0.0008 (10)	−0.0004 (10)	−0.0051 (9)
N2	0.0626 (15)	0.0326 (11)	0.0507 (13)	0.0051 (10)	0.0013 (11)	−0.0040 (10)
C2	0.0482 (15)	0.0410 (14)	0.0448 (14)	0.0018 (12)	0.0021 (12)	−0.0013 (11)
Cl3	0.0585 (5)	0.0442 (4)	0.0767 (5)	−0.0064 (3)	−0.0043 (4)	−0.0129 (3)
C3	0.0626 (19)	0.0423 (15)	0.0440 (15)	0.0078 (13)	0.0044 (13)	0.0068 (11)
N3	0.138 (3)	0.0591 (19)	0.087 (2)	0.036 (2)	−0.001 (2)	0.0074 (16)
N4	0.0297 (11)	0.0483 (13)	0.0576 (13)	0.0067 (9)	−0.0046 (10)	−0.0182 (11)
C4	0.080 (2)	0.0360 (14)	0.0583 (18)	−0.0048 (14)	0.0049 (17)	0.0047 (12)
C5	0.067 (2)	0.0373 (14)	0.0515 (16)	−0.0100 (13)	0.0020 (14)	−0.0023 (12)
C6	0.0457 (14)	0.0339 (13)	0.0441 (14)	−0.0012 (11)	0.0040 (12)	−0.0037 (10)
C7	0.0361 (14)	0.0427 (14)	0.0485 (15)	0.0016 (11)	0.0005 (12)	−0.0111 (12)
C8	0.0557 (18)	0.0526 (17)	0.0526 (16)	0.0086 (14)	−0.0061 (14)	−0.0082 (13)
C9	0.0557 (17)	0.0393 (14)	0.0508 (16)	0.0054 (13)	−0.0012 (13)	−0.0021 (12)
C10	0.086 (2)	0.0515 (18)	0.0538 (17)	0.0120 (17)	−0.0060 (17)	−0.0009 (14)
C11	0.0333 (14)	0.0406 (14)	0.0535 (16)	0.0032 (11)	−0.0002 (12)	−0.0024 (11)
C12	0.0385 (15)	0.0445 (15)	0.0673 (18)	0.0007 (12)	−0.0006 (13)	−0.0132 (13)

### Geometric parameters (Å, º)

**Table:**

O—C11	1.210 (3)	N4—C11	1.335 (3)
Cl1—C2	1.726 (3)	N4—C7	1.402 (3)
C1—C2	1.374 (4)	N4—H4A	0.8600
C1—C6	1.375 (3)	C4—C5	1.378 (4)
C1—H1A	0.9300	C4—H4B	0.9300
Cl2—C3	1.728 (3)	C5—C6	1.384 (4)
N1—N2	1.356 (3)	C5—H5A	0.9300
N1—C7	1.360 (3)	C7—C8	1.348 (4)
N1—C6	1.427 (3)	C8—C9	1.393 (4)
N2—C9	1.330 (3)	C8—H8A	0.9300
C2—C3	1.388 (4)	C9—C10	1.438 (4)
Cl3—C12	1.754 (3)	C11—C12	1.511 (4)
C3—C4	1.376 (4)	C12—H12A	0.9700
N3—C10	1.127 (4)	C12—H12B	0.9700
			
C2—C1—C6	119.7 (2)	C1—C6—C5	121.0 (3)
C2—C1—H1A	120.2	C1—C6—N1	118.8 (2)
C6—C1—H1A	120.2	C5—C6—N1	120.1 (2)
N2—N1—C7	111.4 (2)	C8—C7—N1	107.8 (2)
N2—N1—C6	118.88 (19)	C8—C7—N4	131.1 (2)
C7—N1—C6	129.7 (2)	N1—C7—N4	121.0 (2)
C9—N2—N1	103.7 (2)	C7—C8—C9	104.4 (3)
C1—C2—C3	120.3 (3)	C7—C8—H8A	127.8
C1—C2—Cl1	118.9 (2)	C9—C8—H8A	127.8
C3—C2—Cl1	120.9 (2)	N2—C9—C8	112.7 (2)
C4—C3—C2	119.3 (3)	N2—C9—C10	120.4 (2)
C4—C3—Cl2	119.7 (2)	C8—C9—C10	126.8 (3)
C2—C3—Cl2	121.0 (2)	N3—C10—C9	177.1 (4)
C11—N4—C7	122.4 (2)	O—C11—N4	123.1 (2)
C11—N4—H4A	118.8	O—C11—C12	123.3 (2)
C7—N4—H4A	118.8	N4—C11—C12	113.5 (2)
C3—C4—C5	121.2 (3)	C11—C12—Cl3	111.68 (19)
C3—C4—H4B	119.4	C11—C12—H12A	109.3
C5—C4—H4B	119.4	Cl3—C12—H12A	109.3
C4—C5—C6	118.6 (3)	C11—C12—H12B	109.3
C4—C5—H5A	120.7	Cl3—C12—H12B	109.3
C6—C5—H5A	120.7	H12A—C12—H12B	107.9
			
C7—N1—N2—C9	−1.5 (3)	N2—N1—C7—C8	1.7 (3)
C6—N1—N2—C9	176.8 (2)	C6—N1—C7—C8	−176.4 (3)
C6—C1—C2—C3	−1.6 (4)	N2—N1—C7—N4	−175.1 (2)
C6—C1—C2—Cl1	177.7 (2)	C6—N1—C7—N4	6.9 (4)
C1—C2—C3—C4	1.6 (4)	C11—N4—C7—C8	51.2 (5)
Cl1—C2—C3—C4	−177.7 (2)	C11—N4—C7—N1	−132.9 (3)
C1—C2—C3—Cl2	−179.5 (2)	N1—C7—C8—C9	−1.1 (3)
Cl1—C2—C3—Cl2	1.1 (4)	N4—C7—C8—C9	175.2 (3)
C2—C3—C4—C5	−0.1 (5)	N1—N2—C9—C8	0.8 (3)
Cl2—C3—C4—C5	−178.9 (2)	N1—N2—C9—C10	−179.3 (3)
C3—C4—C5—C6	−1.5 (5)	C7—C8—C9—N2	0.2 (4)
C2—C1—C6—C5	0.1 (4)	C7—C8—C9—C10	−179.7 (3)
C2—C1—C6—N1	−177.2 (2)	N2—C9—C10—N3	176 (8)
C4—C5—C6—C1	1.5 (4)	C8—C9—C10—N3	−4 (9)
C4—C5—C6—N1	178.7 (3)	C7—N4—C11—O	3.5 (5)
N2—N1—C6—C1	35.1 (3)	C7—N4—C11—C12	−175.1 (2)
C7—N1—C6—C1	−147.0 (3)	O—C11—C12—Cl3	2.4 (4)
N2—N1—C6—C5	−142.2 (3)	N4—C11—C12—Cl3	−179.0 (2)
C7—N1—C6—C5	35.7 (4)		

### Hydrogen-bond geometry (Å, º)

**Table:**

*D*—H···*A*	*D*—H	H···*A*	*D*···*A*	*D*—H···*A*
N4—H4*A*···O^i^	0.86	1.95	2.743 (3)	153

Symmetry code: (i) *x*−1, *y*, *z*.
